# Transcriptionally and post-transcriptionally regulated microRNAs in heat stress response in barley

**DOI:** 10.1093/jxb/eru353

**Published:** 2014-09-02

**Authors:** Katarzyna Kruszka, Andrzej Pacak, Aleksandra Swida-Barteczka, Przemyslaw Nuc, Sylwia Alaba, Zuzanna Wroblewska, Wojciech Karlowski, Artur Jarmolowski, Zofia Szweykowska-Kulinska

**Affiliations:** ^1^Department of Gene Expression, Institute of Molecular Biology and Biotechnology, Faculty of Biology, Adam Mickiewicz University in Poznan, Umultowska 89, 61-614 Poznan, Poland; ^2^Bioinformatics Laboratory, Institute of Molecular Biology and Biotechnology, Faculty of Biology, Adam Mickiewicz University in Poznan, Umultowska 89, 61-614 Poznan, Poland

**Keywords:** Barley, heat stress, microRNA, pri-miRNA, splicing, target gene.

## Abstract

Heat stress is one of the major abiotic factors that can induce severe plant damage, leading to a decrease in crop plant productivity. Despite barley being a cereal of great economic importance, few data are available concerning its thermotolerance mechanisms. In this work microRNAs (miRNAs) involved in heat stress response in barley were investigated. The level of selected barley mature miRNAs was examined by hybridization. Quantitative real-time PCR (RT-qPCR) was used to monitor the changes in the expression profiles of primary miRNA (pri-miRNA) precursors, as well as novel and conserved target genes during heat stress. The miRNA-mediated cleavage sites in the target transcripts were confirmed by degradome analysis and the 5’ RACE (rapid amplification of cDNA ends) approach. Four barley miRNAs (miR160a, 166a, 167h, and 5175a) were found which are heat stress up-regulated at the level of both mature miRNAs and precursor pri-miRNAs. Moreover, the splicing of introns hosting miR160a and miR5175a is also heat induced. The results demonstrate transcriptional and post-transcriptional regulation of heat-responsive miRNAs in barley. The observed induction of miRNA expression is correlated with the down-regulation of the expression level of their experimentally identified new and conservative target genes.

## Summary

Selected barley miRNAs and their targets are regulated upon heat stress. Splicing of introns carrying miRNAs was induced by heat and correlated with the accumulation of mature miRNA.

## Introduction

Plants as sessile organisms have developed exceptional abilities to cope with multiple environmental stimuli. The exposure to various stressful environmental conditions requires the induction of proper molecular responses operating at the transcriptional, post-transcriptional, translational, and post-translational levels (Banival *et al*., 2004; Sunkar, 2010). The importance of microRNAs (miRNAs) as key regulators of gene expression at the post-transcriptional level has been widely documented (Mallory and Vaucheret, 2006; Sunkar, 2010; Bielewicz *et al.*, 2013). First identified in *Caenorhabditis elegans* (Lee *et al.*, 1993), miRNAs have been detected in all multicellular eukaryotic organisms (Reinhart *et al.*, 2002; Lagos-Quintana *et al.*, 2003).

MiRNAs are short 20–22 nucleotide (nt) long small RNAs that play a regulatory role in response to environmental stimuli, plant development, or signal transduction (Lu and Huang, 2008; Kruszka *et al.*, 2012; Nozawa *et al.*, 2012). Plant miRNAs are produced from primary (pri-miRNA) precursors that contain a characteristic hairpin structure with the miRNA/miRNA* duplex located within the hairpin stem (Reinhart *et al.*, 2002). The miRNA/miRNA* duplex is diced out from the precursor by a DCL1 enzyme (Kurihara and Watanabe, 2004), which, accompanied mainly by a SERRATE (SE) and a HYPONASTIC LEAVES 1 (HYL1), forms a microprocessing complex (Kurihara *et al.*, 2006; Yang *et al.*, 2006) that ensures efficiency and accuracy of precursor processing to mature miRNA (Dong *et al.*, 2008). Subsequently, the miRNA/miRNA* duplex is exported to the cytoplasm in a HASTY (HST)-dependent manner (Park *et al.*, 2005), where the miRNA guide strand is selectively loaded into an ARGONAUTE1 (AGO1)-containing RNA-induced silencing complex (RISC) (Vaucheret *et al.*, 2004; Baumberger and Baulcombe, 2005). MiRNAs regulate gene expression in a sequence-specific manner (Baulcombe, 2004; Voinnet, 2009) by targeting mRNAs for cleavage or translational repression (Bartel, 2004; Brodersen *et al.*, 2008; Beauclair *et al.*, 2010).

MiRNAs have two major physiological roles in plants: (i) inducing cell differentiation in response to an endogenous stimulus during development and (ii) activation of an adaptive response to a particular exogenous stress. A number of miRNAs have been demonstrated to function in biotic and abiotic stress responses in plants (Sunkar and Zhu, 2004; Khraiwesh *et al.*, 2012; Kruszka *et al.*, 2012; Bielewicz *et al.*, 2013). The role of miRNAs in plants infected by pathogenic bacteria, viruses, nematodes, and fungi has been widely reported (Ruiz-Ferrer and Voinnet, 2009; Katiyar-Agarwal and Jin, 2010). Plant miRNAs have also been reported to play an important role in response to cold (Sunkar and Zhu, 2004; Zhang *et al.*, 2009; Lv *et al.*, 2010), drought (Bartels and Sunkar, 2005; Liu *et al.*, 2008; Jian *et al.*, 2010; Pieczynski *et al.*, 2013), salt (Liu *et al.,* 2008; Zhao *et al.*, 2009), or oxidative stresses induced by heavy metals, salinity, and nutrient deficiency (Sunkar *et al.*, 2006; Jagadeeswaran *et al.*, 2009).

Heat stress is one of the major abiotic stresses that can induce severe plant damage (Baniwal *et al.*, 2004; Wahid *et al.*, 2007). Given the increasing amount of evidence for worldwide climate change, the possible consequences of a temperature increase are a concern not only for climatologists but also for farmers and crop breeders. Heat stress is very often accompanied by drought, and these two combined stressful conditions are the major limitations to food production worldwide, especially in areas that use rainfed agriculture. Observed in recent decades, global warming is predicted to have a general negative effect on plant growth, development, and reproduction mostly due to the destructive effect of high temperature (Bita and Gerats, 2013). The involvement of miRNAs in response to heat has been previously reported in plants such as wheat (Xin *et al.*, 2010), *Brassica rapa* (Yu *et al.*, 2012), *Arabidopsis thaliana* (Guan *et al.*, 2013), and rice (Mittal *et al.*, 2012). Despite the importance of miRNAs, little is known about the mode of regulation of miRNA expression. The importance of the intron within the pri-miRNA 163 and its splicing affecting miRNA biogenesis in *A. thaliana* was shown (Bielewicz *et al.*, 2013; Schwab *et al.*, 2013). No data are available for miRNA biogenesis regulation in monocotyledonous plants.

Barley (*Hordeum vulgare*) grain production ranks fourth in global cereal production (FAO, http://www.fao.org), indicating the economic importance of this monocotyledonous crop plant. For spring barley, an air temperature ~20–25 ºC is considered to be optimum for growth and development. An increase in temperature above these values often results in the reduction of agricultural productivity (Acevedo *et al.*, 2002; Hossain *et al.*, 2012). Hence the importance of determining and understanding the biological factors and processes involved in heat response in plants.

It was considered to be of interest to find out whether barley miRNAs might function in response to high temperature and what might be the mechanisms regulating the miRNA biogenesis upon heat stress. Here it is shown that four mature miRNAs—miR160a, 166a, 167h, and 5175a—are up-regulated under heat stress in barley. The studies also revealed that the level of their pri-miRNAs was affected in the heat stress conditions. Surprisingly, the splicing of the intron-containing precursors pri-miR160a and pri-miR5175a was induced by heat and correlated with the accumulation of mature miRNAs, suggesting the post-trancriptional regulation of miRNA precursor processing. Furthermore, conserved (HD-Zip transcription factors and auxin response factors) as well as novel target genes (*HOX9*, *ACC oxidase*, and *Nek5-like kinase*) of the heat-responsive barley miRNAs were experimentally identified using the 5′ rapid amplification of cDNA ends (RACE) approach and the degradome data. The level of target genes is regulated through a slicing mechanism, and it was experimentally confirmed that the level of the putative target mRNAs was down-regulated in heat stress conditions. A miRNA–target gene network in response to heat stress in barley is discussed. The results of the present study shed new light on the regulation of miRNA precursors and mature miRNA biogenesis, and indicate the important role of miRNAs in response to heat stress in barley.

## Materials and methods

### Plant material and stress conditions

Spring barley plants, cultivar Rolap (Devaux *et al.*, 1992), were grown in a Conviron environmental chamber (Conviron, Winnipeg, Manitoba, Canada) with a 16h day/8h night photoperiod, and 800 μmol light conditions. Plants were grown at 22 ºC day/15 ºC night temperature in 250ml pots containing field soil, and were watered to maintain optimal growth conditions of 70% SWC (soil water content). Two-week-old plants at the three-leaf stage (code 13 of Zadoks system; Zadoks *et al.*, 1974) were subjected to heat treatment at 35.5 ºC. Control plants were grown at a constant temperature of 22 °C. Plants were collected after 3, 6, and 24h of heat stress in three biological replicates. For developmental analyses of expression of selected miRNAs, the whole plants from five growth stages in three biological replicates for each growth stage were used as previously described (Kruszka *et al.*, 2013).

### RNA isolation and northern blots

A 100mg aliquot of tissue was used for isolation of total RNA enriched in small RNA using a protocol as previously described (Pant *et al.*, 2009; Kruszka *et al.*, 2013). The quality and quantity of RNA were measured with a NanoDrop ND-1000 spectrophotometer, and RNA integrity was estimated on agarose gels. RNA electrophoresis, blot transfer, and hybridization were performed as previously reported (Kruszka *et al.*, 2013). DNA oligo probes (Sigma) were 5′ labelled with [γ-^32^P]ATP (6000 Ci mmol^–1^; Hartmann Analytic GmbH, Germany). A DNA probe complementary to U6 small nuclear RNA (snRNA) was used, and the U6 hybridization signal was taken as a loading control. The blots were exposed for 1 week to a phosphorimaging screen (Fujifilm) and scanned with a Fujifilm FLA5100 reader (Fujifilm Co., Ltd, Tokyo, Japan). Blots were quantified with Multi Gauge V2.2 software.

### Amplification of pri-miRNAs using semi-quantitative RT–PCR

RNA for reverse tanscription–PCRs (RT–PCRs) was isolated as described by Kruszka *et al.* (2013). DNA contaminants from the samples were removed using a TURBO DNA-free Kit (Life Technologies, Carlsbad, CA, USA). cDNA templates were synthesized with oligo(dT)_15_ primer (Novazym, Poland) and SuperScript III Reverse Transcriptase (Invitrogen, Carlsbad, CA, USA) using 1 μg of DNase-treated RNA as template. cDNAs from control and stress-treated plants were diluted five times and 2 μl was used for RT–PCR amplification. PCR amplification of a ubiquitin cDNA fragment was used as a positive control. The purity of cDNA samples containing no genomic DNA was controlled by PCR amplification of a barley *phosphate transporter 1* (*HvPht1-1*; GenBank accession no. AF543197.1) promoter fragment (Schumann *et al.*, 2004). Primers were designed for selected expressed sequence tags (ESTs; GenBank accession nos AK361208.1, AK367739.1, AK364148.1, AK362527.1, AK248893.1, AK370571.1, AK374010.1, and AK249660.1) carrying computationally predicted hairpin structure sequences with miRNAs (160a, 166a, 167h, 530-5p, 1120b, 1432-5p, 5175a, and 5203, respectively). The pri-miRNA amplifications were performed with *Taq* DNA polymerase (Thermo Scientific, Lithuania) and two pri-miRNA-specific primers (500nM each) using the thermal profile as previously described (Kruszka *et al.*, 2013). Products of the PCRs were visualized with ethidium bromide staining on 1.2% agarose gels with GeneRuler 100bp Plus (Thermo Scientific) as a length marker. Primer sequences can be found in Supplementary Table S1 available at *JXB* online. PCR products of splicing isoforms were cloned into the pGEM T-Easy vector (Promega, Madison, WI, USA) and sequenced (Faculty’s Laboratory of Molecular Biology Techniques, Adam Mickiewicz University in Poznan, Poland).

### Quantitative real-time PCR (RT-qPCR)

cDNA was prepared and RT-qPCR amplification was performed as previously reported (Kruszka *et al.*, 2013). Expression levels were calculated with the relative quantification method (2^–ΔCt^) as fold change value and presented in the form log_10_2^–ΔCt^. The *R* ^2^ values of analysed data (≥0.997) were calculated with LinRegPCR software (Ramakers *et al.*, 2003). Since the pri-miRNA expression levels are lower than that of the reference gene, the expression profiles are shown in a positive data range without changing the actual values by shifting the zero value of the graph’s *y*-axis to the basal expression level of the whole experiment (Bielewicz *et al.*, 2012). Primers designed and used for the validation of the levels of splicing isoforms were complementary to the exon–intron or exon–exon junctions. Primers are listed in Supplementary Table S1 at *JXB* online.

### Bioinformatics techniques

The sequences of barley miRNA containing cDNAs—160a, 166a, 167h, 530-5p, 1120b, 1432-5p, 5175a, and 5203—were deposited in GenBank (Benson *et al.*, 2012). Sequence analyses and alignments were performed with MAFFT version 6, http://mafft.cbrc.jp/alignment/server/index.html (Katoh and Toh, 2008), and NCBI Blast software, http://blast.ncbi.nlm.nih.gov/Blast.cgi (Altschul *et al.*, 1990). Secondary structures of pre-miRNAs were predicted using Folder Version 1.11 BETA software, http://www.ncrnalab.dk/rnafolder/ with RNAfold, Version 1.6.3 algorithm, http://www.tbi.univie.ac.at/RNA/ (Hofacker *et al.*, 1994). A pri-miRNA fragment covering ~120 nucleotides downstream and upstream of miRNA was used to determine miRNA/miRNA* pairing stability. Structures representing the lowest minimal folding free energy (Δ*G* kcal mol^–1^) are shown herein.

### Target mRNA prediction and cleavage site analysis

The target identification procedure was based on three publicly available programs: miRanda, TAPIR, and psRNAtarget (John *et al.*, 2004; Bonnet *et al.*, 2010; Dai and Zhao, 2011). Full-length cDNA sequences from *H. vulgare* were downloaded from the NCBI and used as the input. Results of the prediction software were subsequently scored based on mismatches present in the predicted sRNA:target duplex alignments where: (i) G:U substitution was counted as 0.5 point; and (ii) other substitutions were given 1 point according to Jones-Rhoades and Bartel (2004) and Schwab *et al.* (2005). No insertions/deletions were allowed. Duplexes having a total mismatch score ≥4 and substitution score in positions 1:12 of sRNA >2.5 points were discarded. For further analysis, only duplexes predicted by at least two programs with MFE energy equal to or lower than –15 kcal mol^–1^ were selected. Protein domain prediction analysis using the HMMER3 tool was performed on putative targets. Protein sequences corresponding to identified cDNAs were extracted using the BLASTx algorithm on the *H. vulgare* protein data set from the NCBI (Altschul *et al.*, 1997; Eddy, 2011). Proteins corresponding to targets with similarity equal to or lower than E-value 0.001 with at least 50% coverage of the known protein were selected. Finally, all putative targets were ranked based on the lowest MFE energy of the duplex and selected according to the position on the list.

To examine cleavage sites of predicted target mRNAs, 5′RACE experiments were conducted with the SMARTer RACE cDNA Amplification Kit (Clontech, Mountain View, CA, USA) according to the manufacturer’s protocol. PCR products were cloned into the pGEM T-Easy vector (Promega) and sequenced (Faculty’s Laboratory of Molecular Biology Techniques, Adam Mickiewicz University in Poznan, Poland).

### Construction and analysis of degradome libraries

A library comprised of 26–27 nt cDNA tags derived from the 5′ ends of polyadenylated RNAs was prepared from poly(A)-enriched RNA from 47- and 68-day-old barley plants. Library construction was similar to previously described methods (Addo-Quaye *et al.*, 2009; German *et al.*, 2009) except that the RNA and DNA adaptors were modified for compatibility to the TruSeq sequencing system of Illumina. The degradome preparation and data analysis will be described in more detail in another publication. The degradome libraries were sequenced by Fasteris SA (Switzerland).

### Accession numbers

The nucleotide sequences of the barley *MIR* genes *MIR160a*, *166a*, *167h*, *530-5p*, *1120b*, *1432-5p*, *5175a*, and *5203* were deposited in GenBank with the respective accession numbers KJ664928, KJ664929, KJ664930, KJ664931, KJ664933, KJ664932, KJ664934, and KJ664935.

## Results

### Mature microRNAs and their precursors are induced by heat stress

To determine the structures of miRNA genes and their transcripts, eight barley cDNA nucleotide sequences for microRNA precursors (160a, 166a, 167h, 530-5p, 1120b, 1432-5p, 5175a, and 5203) deposited in GenBank (http://www.ncbi.nlm.nih.gov/) (Benson *et al.*, 2012) (see the Materials and methods) were selected for which hairpin structures, carrying conserved or newly estimated miRNA homologues, were computationally predicted. First the organization of all barley *MIR* genes was determined. The length and structure for each barley *MIR* gene determined, the nucleotide sequence, and the position of its miRNA/miRNA* within the gene, as well as plant orthologues of barley genes are presented in Table 1. The schematic organization of barley *MIR* genes as well as the miRNA-containing hairpin structures of the barley pre-miRNAs studied, and the orthologues of barley genes in rice, wheat, or *Brachypodium distachyon* are presented in Supplementary Fig. S1 at *JXB* online. Moreover, the expression profiles at the levels of pri-miRNAs and mature miRNAs in five developmental stages are also shown (Supplementary Fig. S2).

**Table 1.** The length and the structure of barley MIR genes

No.	*MIR* gene	Length [bp]	Position of mature miRNA and miRNA* sequences within the gene^*a*^	miRNA sequence 5′→3′	miRNA* sequence 5′→3′	Number of exons [length in bp]	Number of introns [length in bp]	Rice (osa)/wheat (tae)/*Brachypodium (*bdi) orthologues^b^
1	160a	1198	344–364 (intron 2), 415–435*	UGCCUGGCU CCCUGUAUGCCA	GCGUGCAGG AGCCAAGCAUG	4 [206, 47, 99, 348]	3 [73, 211, 214]	tae-miR160
2	166a	1320	174–194 (exon 1), 94–114*	UCGGACCAGGC UUCAUUCCCC	GGAAUGUUGUC UGGUUCAAGG	1 [1320]	0	osa-miR166a
3	167h	1293	119–140 (exon 1), 175–195*	UGAAGCUGCC AGCAUGAUCUGA	AGGUCAUGCUG GAGUUUCAUC	1 [1293]	0	osa-miR167h
4	530–5p	1408	72–92 (exon 1), 171–192*	UGCAUUUGCAC CUGCACCUAC	UAGGUGCAGUG GCAUAUGCAAC	2 [201, 276]	1 [931]	osa-miR530
5	1432–5p	2083	116–136 (exon 1), 188–208*	UUCAGGAGAGA UGACACCGAC	AGGUGUCAUCC CGCCUGAACA	3 [448, 458, 304]	2 [739, 134]	osa-miR1432
6	1120b	2884	1250–1273 (intron 1), 1193–1216*	CUUAUAUUAUGGA ACGGAGGGAGU	UCCCUCCGUCCC AUAAUAUAACAA	3 [242, 35, 173]	2 [2034, 400]	tae-miR1120
7	5175a	5935	5346–5366 (intron 10), 5293–5312*	AAGAAUUUUG GGACGGAGGGA	CCUUCGUCCCAA AAUUCUUGA	11 [857, 139, 52, 1511, 591, 147, 290, 112, 305, 201, 187]	10 [77, 78, 91, 229, 81, 90, 85, 88, 112, 612]	bdi-miR5175a
8	5203	6826	6546–6566, 6477–6497*	ACUUAUUUUGG AACGGAGGGA	ACUCUGCCCUAA AAUAAGUGU	10 [432, 105, 123, 60, 97, 74, 54, 84, 123, 477]	9 [950, 2190, 168, 602, 81, 485, 84, 533, 104]	bdi-miR5180b

^*a*^ The gene nucleotide numbering starts at the first nucleotide of the gene.

^*b*^ Based on nucleotide sequence and hairpin structure similarities, barley *MIR* genes were classified as orthologues of rice, wheat, or *Brachypodium distachyon.*

**Fig. 1.**
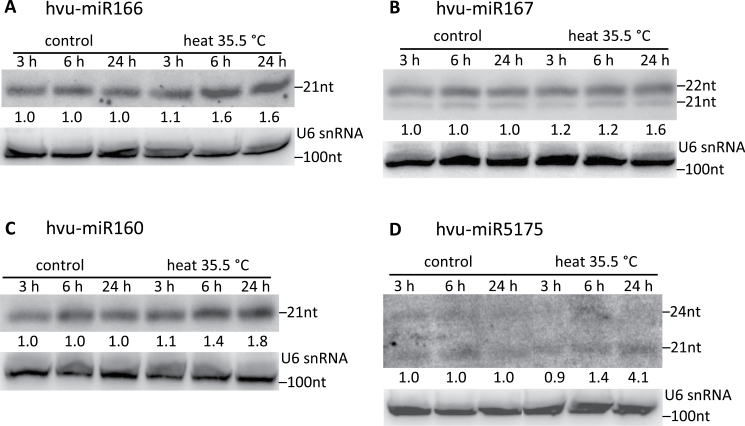
The level of mature barley miRNA166, 160, 167, and 5175 is affected by heat. The mature miRNAs miR166, 167, 160, and 5175 (A–D, respectively) were detected by northern hybridization in control and heat conditions. The level of mature miRNAs was analysed at different time points: 3, 6, and 24h. U6 was used as a loading control. The level of miRNA in heat stress was quantified relative to that in control conditions at the respective time points tested.

The regulation of expression of selected heat-responsive barley miRNAs was studied here. To address this issue, the structure of miRNA genes has to be known. In total the structures of only 16 barley *MIR* genes are available (Kruszka *et al.*, 2013; this study). The accumulation of the mature miRNAs encoded by these genes was examined by northern hybridization (Fig. 1; Supplementary Fig. 3 at *JXB* online). An increase in the mature miRNA level was observed for four mature miRNAs: miR166, 167, 160, and 5175 (Fig. 1). For miR166, a single band of 21 nt in length was detected. Its accumulation was 1.6-fold higher in plants subjected to 6h and 24h heat stress as compared with control plants (Fig. 1A). Northern hybridization with a specific probe for miR167 revealed the presence of two mature miRNAs, 21 nt and 22 nt long, which both were induced in heat-stressed plants (Fig. 1B). The expression level of both miR167 molecules was slightly increased in the case of 3h and 6h heat-treated plants (1.2-fold higher at both time points than in control plants), and was substantially elevated up to 1.6-fold in plants exposed to longer heat treatment. In the case of the mature miR160, its level was increased 1.4- and 1.8-fold in 6h and 24h heat-treated plants, respectively (Fig. 1C). Similarly to the above-described barley miRNAs, the expression level of miR5175 was up-regulated during heat treatment (Fig. 1D). The level of mature miR5175 was particularly elevated in plants exposed to high temperature for a longer period of time (at least 24h). Using the same hybridization probe that was complementary to miR5175, a 24 nt long molecule was also detected. However, the level of accumulation of this molecule did not change in plants subjected to heat stress.

**Fig. 2.**
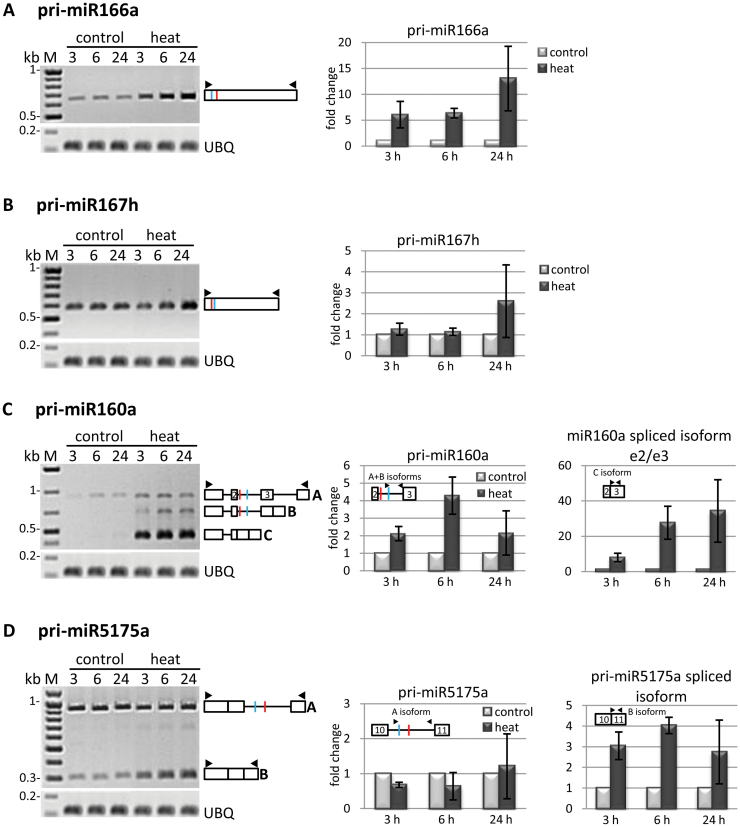
Barley pri-miRNA precursors are induced by heat stress. The panels on the left represent RT–PCR analysis of the expression of pri-miR166a, 167h, 160a, and 5175a (A–D, respectively) in control and heat stress conditions; primer positions are marked by black triangles on the pri-miRNA schematic structures; miRNA and miRNA* are marked in red and blue, respectively. Ubiquitin (UBQ) amplification was used as a loading control. The charts on the right show the real-time PCR measurements of the expression level of pri-miRNA precursors during heat stress. The expression studies were performed at different time points: 3, 6, and 24h. The level of pri-miRNA in control conditions was assumed to be ‘1’, and the levels of pri-miRNA during heat stress were quantified relative to this standard; bars represent the means of three independent biological samples ±SD. M, GeneRuler 100bp Plus or 1kb Plus DNA ladders.

The observed accumulation of mature miRNAs under heat stress raised a question regarding the simultaneous alterations in the level of their primary transcripts as well as putative splicing isoforms. The level of barley pri-miRNAs in heat stress conditions was tested using semi-quantitative RT–PCR. In the case of four miRNA precursors, pri-miR166a, 167h, 160a, and 5175a, considerable changes were also detected in their expression patterns in heat-treated plants, as compared with control plants (Fig. 2, left panels). The results obtained by RT–PCR were confirmed using RT-qPCR (Fig. 2, right panels).

The intronless pri-miR166a accumulated during heat stress and its level increased gradually up to 13-fold after 24h stress as compared with control plants (Fig. 2A, right panel). For the other intronless miR167h primary transcript, the major accumulation was observed in plants stressed for 24h, and its level was 2.6-fold higher than in control conditions (Fig. 2B, right panel). The accumulation of pri-miR166a and pri-miR167h which is accompanied by an increase in their mature miRNAs upon heat stress suggests the transcriptional regulation of the miRNA level.

Interestingly, the high temperature influenced not only the level of the primary transcripts of barley miRNAs but also the level of their spliced isoforms. miR160a is encoded within the second intron of a non-coding transcript containing three introns altogether (Supplementary Fig. S1 at *JXB* online). Pri-miR160a unspliced transcript (isoform A) accumulated considerably in heat-stressed plants compared with control plants (Fig. 2C, left and middle panels). Additionally, two splicing isoforms were observed in heat-treated plants which were not detected in plants grown in control conditions. In the first splicing isoform (isoform B), the last intron was removed, while in the third isoform (isoform C) the last intron as well as the middle, miRNA-containing intron, were spliced. The splicing isoform C, in which intron 2, containing miR160a, was already removed, revealed the most up-regulation in stressed plants, as shown by RT–PCR (Fig. 2C, right panel). Using an RT-qPCR pair of primers specific for the exon 2–exon 3 junction, and exon 3, strong accumulation of this splicing isoform C was confirmed; its level was 8- to 34-fold higher as compared with control plants. Taken together, the mature miR160 up-regulation and accumulation of the unspliced transcript as well as its spliced isoforms in response to heat stress indicate the transcriptional and post-transcriptional regulation of the mature miR160 level.

miR5175a is encoded within the 10th intron of the *RNA polymerase II phosphatase-like/MIR5175a* gene. A similar effect of high temperature on the splicing efficiency of an miRNA-carrying intron was observed for the pri-miR5175a transcript (Fig. 2D, left panel). The level of miR5175a precursor containing intron 10 comprising a miRNA/miRNA* (isoform A) was slightly decreased in plants after 3h and 6h of heat stress, while in plants after 24h of stress its level was similar to that of control plants. This observation was confirmed by RT-qPCR using primers specific for intron 10 in which miR5175a is located (Fig. 2D, middle panel). Simultaneously, a substantial accumulation of the splicing isoform lacking intron 10 (isoform B) in stress-treated plants was observed. RT-qPCR amplification with a specific pair of primers anchored in the exon 10–exon 11 junction and exon 11 confirmed strong accumulation of the spliced isoform whose level was 4-fold higher as compared with control plants (Fig. 2D, right panel). These results implicate the post-transcriptional regulation of miR5175a expression.

The described results show an unusual observation that heat-induced splicing and splicing efficiency of the miRNA-bearing introns studied correlate with mature miRNA accumulation.

### Elevated expression of barley heat-regulated miRNAs correlates with down-regulation of the expression of their target genes

To understand the function of barley miRNAs in response to heat stress, the level of putative miRNA-regulated target mRNAs was investigated. The expression level of target mRNAs was estimated by RT-qPCR. The 5′RACE experiments as well as the degradome sequencing were carried out to verify the predicted cleavage sites in target mRNAs.

**Fig. 3.**
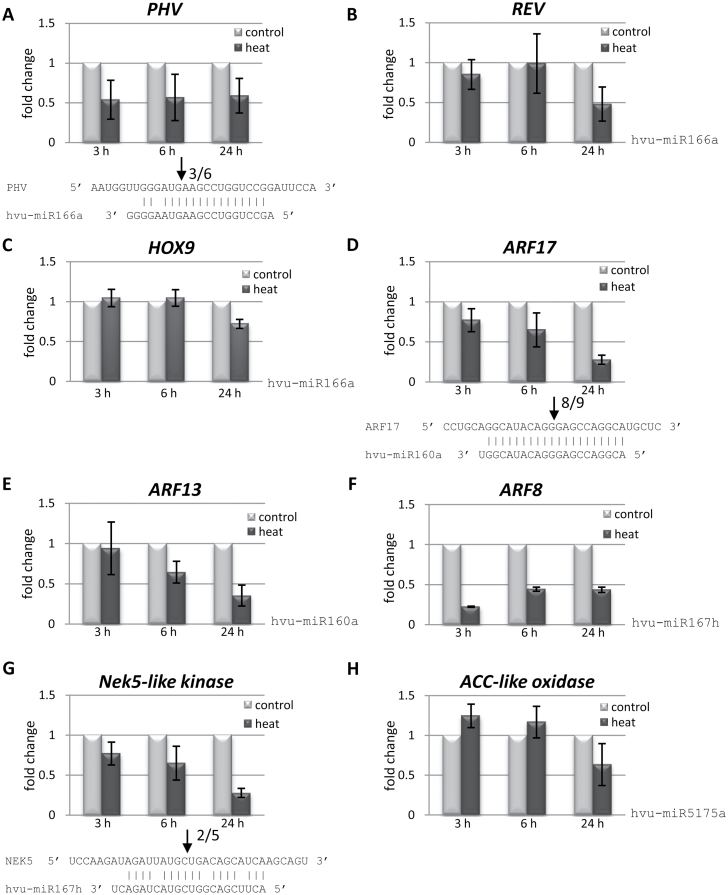
Heat stress affects the expression level of barley miRNA-regulated target genes. RT-qPCR analysis was performed to examine the expression level of target transcripts sliced by: miR166a, *PHV* (A), *REV* (B), and *HOX9* (C); miR160a, *ARF17* (D) and *ARF13* (E); miR167h, *ARF8* (F) and *Nek5* (G); and miR5175a, *ACC-like oxidase* (H). mRNA:miRNA base-pairing diagrams below the graphs for *PHV*, *AFR17*, and *Nek5* transcripts (A, D, and G, respectively) show the slicing sites. Arrows, along with the number of clones analysed, indicate the 5’ end of the cleaved product validated by 5’RACE.

**Fig. 4.**
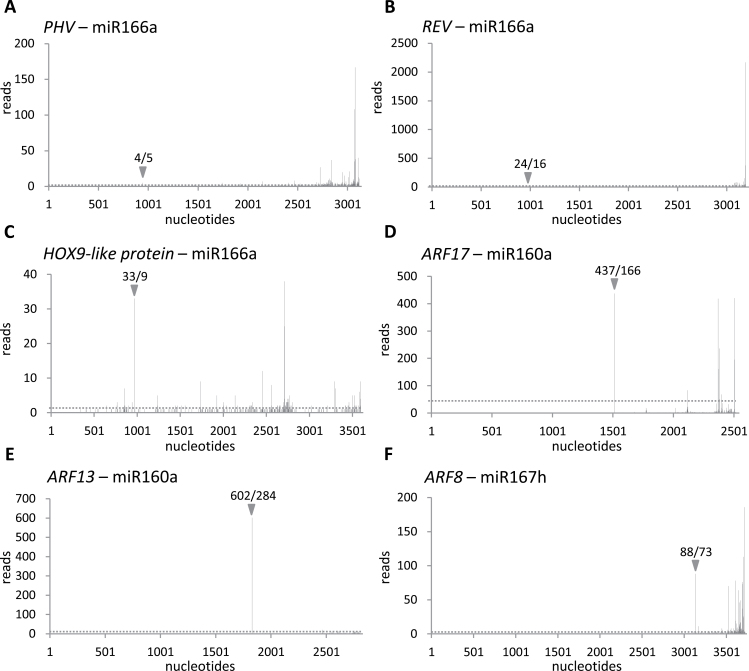
Target plots (t-plots) for barley miRNA target detection using data from degradome libraries. t-plots show the distribution of the degradome tag reads along the full length of the target mRNA sequence. Grey arrows, along with the number of tag reads identified in the degradome libraries from 6-week- and 68-day-old barley plants, respectively, indicate the cleavage sites guided by miR166a (A, B, C), 160a (D, E), and 167h (F). The dotted line represents the average read value for the entire mRNA sequence.

In *A. thaliana*, miR166a is known to target mRNAs coding for HD-Zip transcription factors including PHAVOLUTA (PHV), which regulates auxiliary meristem initiation and leaf development (Rhoades *et al.*, 2002). A barley orthologue of the *Arabidopsis PHV* gene (GenBank accession no. AK364215.1) was analysed as a potential target candidate. The expression level of *PHV* mRNA decreased nearly 2-fold in heat-stressed plants at all time points tested as compared with control plants. Using 5′RACE and degradome data, the cleavage site in the *PHV* transcript guided by miR166a was confirmed (Figs 3A, 4A). The *REVOLUTA* gene (*REV*; GenBank accession no. AK362009) is the other member of the HD-Zip transcription factor family predicted to be a putative target mRNA of barley miR166a. The expression level of the *REV* transcript was also reduced under heat stress, mostly after 24h exposure to heat. The degradome data confirmed the predicted slicing site (Figs 3B, 4B). A *homeobox-leucine zipper protein HOX9-like* gene (GenBank accession no. AK354023.1) was also identified as a novel miR166a target gene in barley. The HOX9 protein is another member of the HD-Zip transcription factor family which was reported to regulate rice embryogenesis (Nagasaki *et al.*, 2007). The expression level of *HOX9* mRNA was decreased especially after 6h and 24h of heat stress, and the predicted slicing site guided by miR166a was confirmed by the degradome data (Figs 3C, 4C).

mRNAs encoding the auxin response transcription factor *ARF17* (GenBank accession no. AK363500.1) and *ARF13* (GenBank accession no. AK364144) were confirmed as conserved miR160 targets in barley. In *Arabidopsis thaliana* miR160 was reported to target *ARF* genes (Rhoades *et al.*, 2002). The level of barley *ARF17* and *ARF13* transcripts was considerably down-regulated to a 4-fold lower level in heat stress conditions than in control plants, especially in plants exposed to elevated temperature for 24h (Fig. 3D, E). The cleavage site guided by miR160a in ARF17 mRNA was verified by the 5′RACE approach. In addition, the predicted cleavage sites in both *ARF17* and *ARF13* transcripts were confirmed by the degradome data (Fig. 4D, E).

Two potential mRNA targets of miR167h were predicted—an *auxin response transcription factor ARF8* gene (GenBank accession no. AK368822.1) and a *serine/threonine-protein kinase Nek5-like* gene (GenBank accession no. AK363207) (Fig. 3F, G). In *Arabidopsis* and rice, the members of the auxin response transcription factor family were previously reported as miR167 family targets (Jones-Rhoades and Bartel, 2004). In the present studies it was shown that barley *ARF8* mRNA is strongly down-regulated under heat stress (Fig. 3F). However, it was not possible to map the miR167h-guided cleavage site of the *ARF8* transcript precisely as all examined 5′RACE clones were at least 35 nt shorter than the expected 3′ cleavage products. The lack of precisely sliced 3′ products was most probably due to rapid degradation of the cleaved fragments of the *ARF8* transcript. However, it was determined that the strong peak from the degradome library sequencing data corresponded precisely to the predicted cleavage site (Fig. 4F). A Nek5-like gene transcript was identified as a novel target gene of barley miR167h (Fig. 3G). The mRNA level of the *NEK5-like kinase* gene decreased considerably in plants exposed to 24h heat stress. The cleavage site in the *NEK5* transcript that is recognized by miR167h was also verified using the 5′RACE approach; however, it was not possible to detect the slicing site using the degradome data.

In the case of miR5175a, a novel putative target mRNA was predicted—an enzyme involved in ethylene biosynthesis ACC-like oxidase (GenBank accession no. AK370060.1) (Raz and Ecker, 1999). The expression level of *ACC-like oxidase* mRNA was down-regulated mainly in 24h heat-stressed plants (Fig. 3H). However, it was not possible to detect slicing products either in 5′RACE or in the degradome sequencing data.

The results show the conservation of the target mRNAs between barley and dicot plants and reveal the existence of new additional, as yet unrecognized target mRNAs.

## Discussion

Barley *MIR* genes show a diverse genomic organization, as previously reported (Kruszka *et al.*, 2013). Most of the *MIR* genes in this study contain at least one intron. Interestingly, some members of the same family were encoded by genes that differ noticeably in structure, as was revealed for the barley *MIR166* family. A similar observation has been described for members of the *MIR160* family in *Arabidopsis* (Szarzynska *et al.*, 2009). These observations may indicate that miRNAs encoded by the different genes representing the same *MIR* gene family might differ in their expression regulation pattern at the post-transcriptional level. The intron-containing pri-miRNA transcripts investigated herein undergo a complex splicing pattern. This phenomenon, previously described for both *Hordeum* and *Arabidopsis* miRNA precursors (Szarzynska *et al.*, 2009; Kruszka *et al.*, 2013), may suggest a potential regulatory role of splicing in mature miRNA biogenesis. This suggestion has been recently confirmed for *Arabidopsis* miRNA precursors (Bielewicz *et al.*, 2013; Schwab *et al.*, 2013). The presence of an intron in pri-miRNA transcripts can promote mature miRNA accumulation, through a mechanism that probably operates at the level of miRNA processing or stability.

Remarkably, not only were intron-containing pri-miRNAs undergoing splicing detected, but, more importantly, the splicing events were observed to be strongly affected in barley plants subjected to heat stress. The findings suggest the post-transcriptional regulation of the expression of these two barley miRNAs. Surprisingly, substantial accumulation of miR160a and miR5175a splicing isoforms was observed, mostly lacking introns which contain the miRNA-bearing hairpin structures. Previous studies on pre-mRNA splicing have indicated the shutdown of pre-mRNA splicing in response to heat stress (Yost and Lindquist, 1986; Bond, 1988; Shin *et al.*, 2004). Moreover, in plants, it has been previously shown that insertion of an artificial secondary structure (stem–loop) in an intron strongly inhibits splicing *in vivo* in tobacco protoplasts (Goodall and Filipowicz, 1991). A low level of barley pri-miRNAs splicing in control plants was observed, which might suggest the inhibitory role of stem–loop structures in intron splicing. However, the presence of such secondary structure might also modulate the splicing in response to different conditions such as heat stress. RNA structures are highly sensitive to the concentration and types of ions and osmolytes present (Draper, 2004; Lambert and Draper, 2007; Mullen *et al.*, 2012). It is known that many abiotic stresses, such as high salinity, heat, and drought, impact cellular concentrations of ions and osmolytes in plants (Seki *et al.*, 2007; Mullen *et al.*, 2012; Chan *et al.*, 2013), which might impact RNA secondary structure and regulate splicing and other aspects of post-transcriptional gene regulation. Hence, structural elements in pri-miRNAs might function as sensors of abiotic stresses, such as temperature, drought, and salinity (Reddy *et al.*, 2013).

In the case of intronless *MIR166a* and *MIR167* genes, an increase in the level of both mature miRNAs and their precursors was detected upon heat stress. These results strongly suggest the transcriptional regulation of the expression of these miRNAs; however, the effect of pri-miRNA transcript stability cannot be excluded.

The present study has shown that barley miRNA precursors as well as mature miRNA molecules were heat inducible, which might be required for barley thermotolerance through down-regulation of their target genes. The involvement of plant miRNAs in response to high temperature was previously shown in plants such as *Arabidopsis* (Wang *et al.*, 2012; Guan *et al.*, 2013), *Populus tomentosa* (Chen *et al.*, 2012), or *Brassica rapa* (Yu *et al.*, 2012). Interestingly, the high-throughput sequencing of the wheat miRNAome allowed identifiction of a set of eight miRNAs that were up-regulated in heat stress conditions, including tae-miR160, 166, and 167 (Xin *et al.*, 2010). This finding is consistent with the present results; however, miR160 is strongly down-regulated during heat stress in *Populus* (Chen *et al.*, 2012). These results suggest the existence of a common set of miRNAs involved in response to heat in plants, though the response of miRNAs might differ significantly depending on the species studied. The transcriptome analysis of wheat revealed a large number of heat stress-responsive genes encoding transcription factors and proteins involved in phytohormone biosynthesis/signalling, calcium and sugar signalling pathways, RNA metabolism, ribosomal proteins, primary and secondary metabolism, as well as proteins related to other stresses (Qin *et al.*, 2008). However, only a few of the identified heat stress-responsive genes were tested and confirmed as being regulated by miRNAs in heat stress conditions (Xin *et al.*, 2010).

When the level of a miRNA is up-regulated, usually its target gene is down-regulated post-transcriptionally. In the present study, the conserved and the novel targets of barley miRNAs that function in diverse pathways and processes were identified. Barley as a crop plant is sensitive to high temperature, especially during the grain-filling stage (Hakala *et al.*, 2012). Frequently identified features of plants exposed to high temperatures are a significant decrease in height and biomass reduction. In addition, heat stress which occurs during the early stages of barley development is known to reduce tillering, thereby decreasing crop yield (Savin *et al.*, 1997; Wallwork *et al.*, 1998; Alam *et al.*, 2007). Considering barley growth and development, heat stress was reported to decrease the days to visible awns, days to heading, and days to ripe harvest (Wallwork *et al.*, 1998). Heat stress not only affects the aerial parts of the plants but also influences the growth and morphology of the roots, causing the arrest of root elongation and branching (Marschner, 1995).

**Fig. 5.**
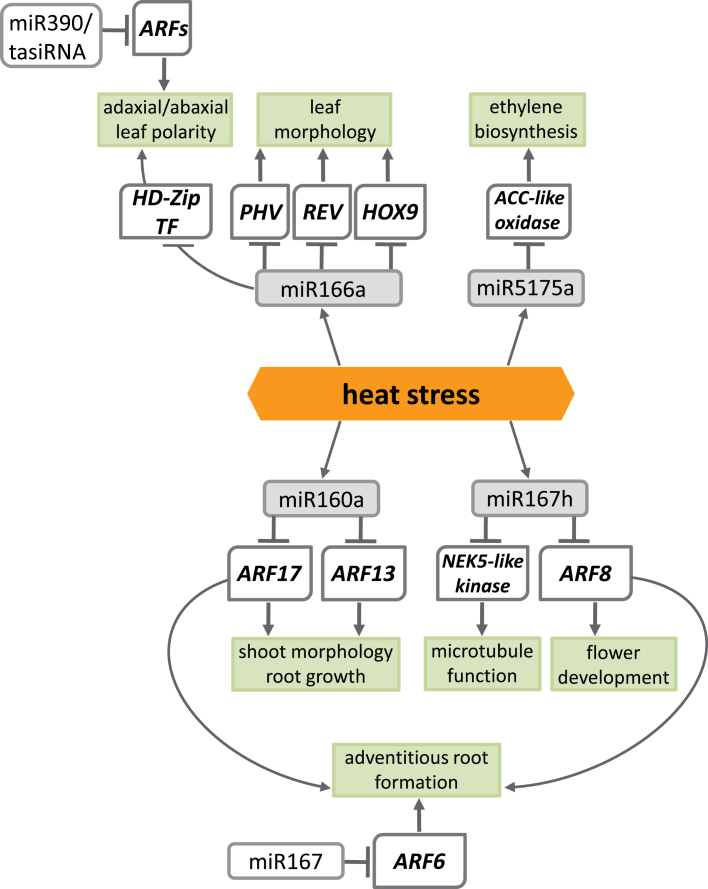
An miRNA–target genes network module involved in heat stress response in barley.

The module of the putative network of heat-responsive barley miRNAs and their respective target genes involved in the heat stress response is presented in Fig. 5. *PHAVOLUTA*, *REVOLUTA*, and *HOX9* are the HD-Zip transcription factors whose expression is regulated by miR166a, an evolutionarily conserved plant miRNA (Barik *et al.*, 2014). These transcription factors have been shown to be required for the establishment of the apical meristem, proper pattern formation in lateral organs, and leaf development (Rhoades *et al.*, 2002; Nagasaki *et al.*, 2007). In plants such as maize and *Arabidopsis*, the adaxial/abaxial leaf polarity is established by fine-tuned interplay between an abaxial gradient of miR166, adaxial presence of a ta-siRNA (trans-acting small interfering RNA), and their targets (Juarez *et al.*, 2004; Nogueira *et al.*, 2007; Husbands *et al.*, 2009). Thus, the changes in the level of miR166 in heat stress might influence the leaf morphology. Shoot morphology and regeneration *in vitro* are also affected by changes in the miR160a level (Qiao *et al.*, 2012). It was determined in this study that an increase in barley miR160a during heat stress considerably down-regulates the expression level of the auxin response transcription factors *ARF17* and *ARF13*. ARF17 is a regulator of GH3-like early auxin response genes, and its function was reported in emerging leaf symmetry, premature inflorescence development, and flower development (Mallory *et al.*, 2005). Auxin is an important hormone involved in many plant processes including cell elongation and division, and thus growth (Overvoorde *et al.*, 2010; Gallavotti, 2013; Sauer *et al.*, 2013; de Wit *et al.*, 2014). As the heat stress effects on plants often include growth inhibition, the auxin signalling pathway might be an important component of plant thermotolerance.

It was also confirmed that another auxin-responsive transcription factor gene, *ARF8*, a target gene of miR167h, was down-regulated by heat in barley. In *Arabidopsis*, *ARF8* regulates floral organ development and vascular patterning of petals (Tabata *et al.*, 2010). Moreover, *ARF8* together with *AFR6* and *ARF17* regulates adventitious root formation in *Arabidopsis* (Gutierrez *et al*., 2009, 2012). Furthermore, the involvement of miR160 and miR167 families targeting *ARF* transcription factors in lateral and adventitious root formation, as well as root cap development, has been shown in rice (Meng *et al.*, 2009, 2010). The three *ARF* genes display overlapping expression domains, interact genetically, and regulate each other’s expression at both the transcriptional and post-transcriptional level by modulating miR160 and miR167 availability (Gutierrez *et al.*, 2009). In addition, the fine-tuning of adventitious root initiation requires the *GH3* genes that modulate jasmonic acid homeostasis (Gutierrez *et al.*, 2012). As root growth inhibition is one of the major consequences of the effect of high temperature on plants, it is of great importance to understand this cross-talk between plant hormones, transcription factors, and miRNAs.

Interestingly, a novel target gene of barley miR167h, *Nek5-like kinase*, was also identified in the present study. In *Arabidopsis*, the NEK protein subfamily, including NEK4, NEK5, and NEK6, plays an important role in epidermal cell expansion and microtubule functions (Motose *et al.*, 2011). It has been reported by Nakajima and Suzuki (2013) that heat stress induces misfolded protein aggregation in plant cells in a microtubule-dependent manner. In addition, a link between miRNA activities and microtubules was also observed in plants and animals, suggesting that miRNA-based translational repression or miRNA activities in general may rely on cytoskeleton dynamics in diverse species (Ma *et al.*, 2013).

In conclusion, the present study revealed that four barley miRNAs—miR160a, 166a, 167h, and 5175a—respond to heat stress. The expression level of barley pri-miRNAs as well as the splicing of miRNA-bearing introns was induced in plants subjected to heat stress, suggesting the transcriptional as well post-transcriptional regulation of miRNA expression. The accumulation of miRNAs triggered the changes in the expression level of conserved and novel target genes characterized in this study. The findings showed that barley miRNAs, together with target genes whose expression they regulate, might function in a complex regulatory network developed by barley to cope with stressful conditions. The findings will contribute to explaining the mechanisms of plant thermotolerance.

## Supplementary data

Supplementary data are available at *JXB* online.

Figure S1. Schematic representation of barley *MIR* genes and the hairpin structures of barley/plant orthologues of the pre-miRNA precursors.

Figure S2. The expression profiles at the level of pri-miRNAs and mature miRNAs during barley development.

Figure S3. The level of mature barley miRNAs under heat stress.

Figure S4. Expression of the heat stress marker gene *HSP17* in barley plants subjected to heat stress.

Table S1. Primers and hybridization probes used in the experiments.

Supplementary Data

## References

[CIT0001] Acevedo E Silva P Silva H. 2002. Wheat growth and physiology. In: Curtis BC, ed. Bread wheat: improvement and production. FAO Plant Production and Protection Series No. 30. Rome: FAO, 567.

[CIT0002] Addo-Quaye C Snyder JA Park YB Li YF Sunkar R Axtell MJ. 2009. Sliced microRNA targets and precise loop-first processing of *MIR319* hairpins revealed by analysis of the *Physcomitrella patens* degradome. RNA 15, 2112–2121 19850910 10.1261/rna.1774909 PMC2779683

[CIT0003] Alam MZ Haider SA Paul NK. 2007. Yield and yield components of barley (*Hordeum vulgare* L.) in relation to sowing times. Journal of Bio-Science 15, 139–145

[CIT0004] Altschul SF Gish W Miller W Myers EW Lipman DJ. 1990. Basic local alignment search tool. Journal of Molecular Biology 215, 403–410 2231712 10.1016/S0022-2836(05)80360-2

[CIT0005] Altschul SF Madden TL Schäffer AA Zhang J Zhang Z Miller W Lipman DJ. 1997. Gapped BLAST and PSI-BLAST: a new generation of protein database search programs. Nucleic Acids Research 25, 3389–3402 9254694 10.1093/nar/25.17.3389 PMC146917

[CIT0006] Baniwal SK Bharti K Chan KY. 2004. Heat stress response in plants: a complex game with chaperones and more than twenty heat stress transcription factors. Journal of Biosciences 29, 471–487 15625403 10.1007/BF02712120

[CIT0007] Barik S Sarkardas S Singh A Gautam V Kumar P Majee M Sarkar AK. 2014. Phylogenetic analysis reveals conservation and diversification of micro RNA166 genes among diverse plant species. Genomics 103, 114–121 24275521 10.1016/j.ygeno.2013.11.004

[CIT0008] Bartel DP. 2004. MicroRNAs: genomics, biogenesis, mechanism, and function. Cell 116, 281–297 14744438 10.1016/s0092-8674(04)00045-5

[CIT0009] Bartels D Sunkar R. 2005. Drought and salt tolerance in plants. Critical Reviews in Plant Sciences 24, 23–58

[CIT0010] Baulcombe D. 2004. RNA silencing in plants. Nature 431, 356–363 15372043 10.1038/nature02874

[CIT0011] Baumberger N Baulcombe DC. 2005. *Arabidopsis* ARGONAUTE1 is an RNA Slicer that selectively recruits microRNAs and short interfering RNAs. Proceedings of the National Academy of Sciences, USA 102, 11928–11933 10.1073/pnas.0505461102 PMC1182554 16081530

[CIT0012] Beauclair L Yu A Bouche N. 2010. microRNA-directed cleavage and translational repression of the copper chaperone for superoxide dismutase mRNA in Arabidopsis. The Plant Journal 62, 454–462 20128885 10.1111/j.1365-313X.2010.04162.x

[CIT0013] Benson DA Karsch-Mizrachi I Clark K Lipman DJ Ostell J Sayers EW. 2012. GenBank. Nucleic Acids Research 40, D48–D53 22144687 10.1093/nar/gkr1202 PMC3245039

[CIT0014] Bielewicz D Dolata J Zielezinski A Alaba S Szarzynska B Szczesniak MW Jarmolowski J Szweykowska-Kulinska Z Karlowski W. 2012. mirEX: a platform for comparative exploration of plant pri-miRNA expression data. Nucleic Acids Research 40, 191–197 10.1093/nar/gkr878 PMC3245179 22013167

[CIT0015] Bielewicz D Kalak M Kalyna M Windels D Barta A Vazquez F Szweykowska-Kulinska Z Jarmolowski A. 2013. Introns of plant pri-miRNAs enhance miRNA biogenesis. EMBO Reports 14, 622–628 23681439 10.1038/embor.2013.62 PMC3701235

[CIT0016] Bita CE Gerats T. 2013. Plant tolerance to high temperature in a changing environment: scientific fundamentals and production of heat stress-tolerant crops. Frontiers in Plant Science 4, 273 23914193 10.3389/fpls.2013.00273 PMC3728475

[CIT0017] Bond U. 1988. Heat shock but not other stress inducers leads to the disruption of a sub-set of snRNPs and inhibition of *in vitro* splicing in HeLa cells. EMBO Journal 7, 3509–3518 2974799 10.1002/j.1460-2075.1988.tb03227.x PMC454852

[CIT0018] Bonnet E He Y Billiau K Van de Peer Y. 2010. TAPIR, a web server for the prediction of plant microRNA targets, including target mimics. Bioinformatics 26, 1566–1568 20430753 10.1093/bioinformatics/btq233

[CIT0019] Brodersen P Sakvarelidze-Achard L Bruun-Rasmussen M Dunoyer P Yamamoto YY Sieburth L Voinnet O. 2008. Widespread translational inhibition by plant miRNAs and siRNAs. Science 320, 1185–1190 18483398 10.1126/science.1159151

[CIT0020] Chan KX Wirtz M Phua SY Estavillo GM Pogson BJ. 2013. Balancing metabolites in drought: the sulfur assimilation conundrum. Trends in Plant Science 18, 18–29 23040678 10.1016/j.tplants.2012.07.005

[CIT0021] Chen L Ren Y Zhang Y Xu J Sun F Zhang Z Wang Y. 2012. Genome-wide identification and expression analysis of heat-responsive and novel microRNAs in *Populus tomentosa* . Gene 504, 160–165 22634103 10.1016/j.gene.2012.05.034

[CIT0022] Dai X Zhao PX. 2011. psRNATarget: a plant small RNA target analysis server. Nucleic Acids Research 39, W155–W159 21622958 10.1093/nar/gkr319 PMC3125753

[CIT0023] Devaux P Adamski T Surma M. 1992. Inheritance of seed set in crosses of spring barley and *Hordeum bulbosum* L. Crop Science 32, 269–271

[CIT0024] de Wit M Lorrain S Fankhauser C. 2014. Auxin-mediated plant architectural changes in response to shade and high temperature. Physiologia Plantarum 151, 13–24 24011166 10.1111/ppl.12099

[CIT0025] Dong Z Han M-H Fedoroff N. 2008. The RNA-binding proteins HYL1 and SE promote accurate *in vitro* processing of pri-miRNA by DCL1. Proceedings of the National Academy of Sciences, USA 105, 9970–9975 10.1073/pnas.0803356105 PMC2481344 18632569

[CIT0026] Draper DE. 2004. A guide to ions and RNA structure. RNA 10, 335–343 14970378 10.1261/rna.5205404 PMC1370927

[CIT0027] Eddy SR. 2011. Accelerated profile HMM searches. PLoS Computational Biology 7, e1002195 22039361 10.1371/journal.pcbi.1002195 PMC3197634

[CIT0028] Gallavotti A. 2013. The role of auxin in shaping shoot architecture. Journal of Experimental Botany 64, 2593–2608 23709672 10.1093/jxb/ert141

[CIT0029] German MA Luo S Schroth G Meyers BC Green PJ. 2009. Construction of parallel analysis of RNA ends (PARE) libraries for the study of cleaved miRNA targets and the RNA degradome. Nature Protocols 4, 356–362 10.1038/nprot.2009.8 19247285

[CIT0030] Goodall GJ Filipowicz W. 1991. Different effects of intron nucleotide composition and secondary structure on pre-mRNA splicing in monocot and dicot plants. EMBO Journal 10, 2635–2644 1868837 10.1002/j.1460-2075.1991.tb07806.x PMC452964

[CIT0031] Guan Q Lu X Zeng H Zhang Y Zhu J. 2013. Heat stress induction of *miR398* triggers a regulatory loop that is critical for thermotolerance in Arabidopsis. The Plant Journal 74, 840–851 23480361 10.1111/tpj.12169

[CIT0032] Gutierrez L Bussell JD Pacurar DI Schwambach J Pacurar M Bellini C. 2009. Phenotypic plasticity of adventitious rooting in Arabidopsis is controlled by complex regulation of AUXIN RESPONSE FACTOR transcripts and microRNA abundance. The Plant Cell 21, 3119–3132 19820192 10.1105/tpc.108.064758 PMC2782293

[CIT0033] Gutierrez L Mongelard G Floková K. 2012. Auxin controls *Arabidopsis* adventitious root initiation by regulating jasmonic acid homeostasis. The Plant Cell 24, 2515–2527 22730403 10.1105/tpc.112.099119 PMC3406919

[CIT0034] Hakala K Jauhiainen L Himanen SJ Rötter R Salo T Kahiluoto H. 2012. Sensitivity of barley varieties to weather in Finland. Journal of Agricultural Science 150, 145–160 22505777 10.1017/S0021859611000694 PMC3320810

[CIT0035] Hofacker IL Fontana W Stadler PF Bonhoeffer LS Tacker M Schuster P. 1994. Fast folding and comparison of RNA secondary structures (The Vienna RNA Package). Monatshefte fűr Chemie 125, 167–188

[CIT0036] Hossain A Teixeira da Silva JA Lozovskaya MV Zvolinsky VP. 2012. High temperature combined with drought affect rainfed spring wheat and barley in South-Eastern Russia: I. Phenology and growth. Saudi Journal of Biological Sciences 19, 473–487 23961209 10.1016/j.sjbs.2012.07.005 PMC3730755

[CIT0037] Husbands AY Chitwood DH Plavskin Y Timmermans MC. 2009. Signals and prepatterns: new insights into organ polarity in plants. Genes and Development 23, 1986–1997 19723761 10.1101/gad.1819909 PMC2751976

[CIT0038] Jagadeeswaran G Saini A Sunkar R. 2009. Biotic and abiotic stress down-regulate miR398 expression in *Arabidopsis* . Planta 229, 1009–1014 19148671 10.1007/s00425-009-0889-3

[CIT0039] Jian X Zhang L Li G Zhang L Wang X Cao X Fang X Chen F. 2010. Identification of novel stress regulated microRNAs from *Oryza sativa* L. Genomics 95, 47–55 19796675 10.1016/j.ygeno.2009.08.017

[CIT0040] John B Enright AJ Aravin A Tuschl T Sander C Marks DS. 2004. Human microRNA targets. PLoS Biology 2, e363 15502875 10.1371/journal.pbio.0020363 PMC521178

[CIT0041] Jones-Rhoades MW Bartel DP. 2004. Computational identification of plant microRNAs and their targets, including a stress-induced miRNA. Molecular Cell 14, 787–799 15200956 10.1016/j.molcel.2004.05.027

[CIT0042] Juarez MT Kui JS Thomas J Heller BA Timmermans MC. 2004. microRNA-mediated repression of *rolled leaf1* specifies maize leaf polarity. Nature 428, 84–88 14999285 10.1038/nature02363

[CIT0043] Katiyar-Agarwal S Jin H. 2010. Role of small RNAs in host–7microbe interactions. Annual Review of Phytopathology 48, 225–246 10.1146/annurev-phyto-073009-114457 PMC3752435 20687832

[CIT0044] Katoh K Toh H. 2008. Recent developments in the MAFFT multiple sequence alignment program. Briefings in Bioinformatics 9, 286–298 18372315 10.1093/bib/bbn013

[CIT0045] Khraiwesh B Zhua JK Zhuc J. 2012. Role of miRNAs and siRNAs in biotic and abiotic stress responses of plants. Biochimica et Biophysica Acta 1819, 137–148 21605713 10.1016/j.bbagrm.2011.05.001 PMC3175014

[CIT0046] Kruszka K Pacak A Swida-Barteczka A Stefaniak AK Kaja E Sierocka I Karlowski W Jarmolowski A Szweykowska-Kulinska Z. 2013. Developmentally regulated expression and complex processing of barley pri-microRNAs. BMC Genomics 14, 34 23324356 10.1186/1471-2164-14-34 PMC3558349

[CIT0047] Kruszka K Pieczynski M Windels D Bielewicz D Jarmolowski A Szweykowska-Kulinska Z Vazquez F. 2012. Role of microRNAs and other sRNAs of plants in their changing environments. Journal of Plant Physiology 169, 1664–1672 22647959 10.1016/j.jplph.2012.03.009

[CIT0048] Kurihara Y Takashi Y Watanabe Y. 2006. The interaction between DCL1 and HYL1 is important for efficient and precise processing of pri-miRNA in plant microRNA biogenesis. RNA 12, 206–212 16428603 10.1261/rna.2146906 PMC1370900

[CIT0049] Kurihara Y Watanabe Y. 2004. *Arabidopsis* micro-RNA biogenesis through Dicer-like 1 protein functions. Proceedings of the National Academy of Sciences, USA 101, 12753–12758 10.1073/pnas.0403115101 PMC515125 15314213

[CIT0050] Lagos-Quintana M Rauhut R Meyer J Borkhardt A Tuschl T. 2003. New microRNAs from mouse and human. RNA 9, 175–179 12554859 10.1261/rna.2146903 PMC1370382

[CIT0051] Lambert D Draper DE. 2007. Effects of osmolytes on RNA secondary and tertiary structure stabilities and RNA–Mg^2+^ interactions. Journal of Molecular Biology 370, 993–1005 17555763 10.1016/j.jmb.2007.03.080 PMC1995082

[CIT0052] Lee RC Feinbaum RL Ambros V. 1993. The C. elegans heterochronic gene *lin-4* encodes small RNAs with antisense complementarity to *lin-14* . Cell 75, 843–854 8252621 10.1016/0092-8674(93)90529-y

[CIT0053] Liu HH Tian X Li YJ Wu CA Zheng CC. 2008. Microarray-based analysis of stress-regulated microRNAs in *Arabidopsis thaliana* . RNA 14, 836–843 18356539 10.1261/rna.895308 PMC2327369

[CIT0054] Lu HY Huang XL. 2008. Plant miRNAs and abiotic stress responses. Biochemical and Biophysical Research Communications 368, 458–462 18267107 10.1016/j.bbrc.2008.02.007

[CIT0055] Lv DK Bai X Li Y Ding XD Ge Y Cai H Ji W Wu N Zhu YM. 2010. Profiling of cold-stress-responsive miRNAs in rice by microarrays. Gene 459, 39–47 20350593 10.1016/j.gene.2010.03.011

[CIT0056] Ma X Cao X Mo B Chen X. 2013. Trip to ER: microRNA-mediated translational repression in plants. RNA Biology 10, 1586–1592 24100209 10.4161/rna.26313 PMC3866237

[CIT0057] Mallory AC Bartel DP Bartel B. 2005. MicroRNA-directed regulation of Arabidopsis *AUXIN RESPONSE FACTOR17* is essential for proper development and modulates expression of early auxin response genes. The Plant Cell 17, 1360–1375 15829600 10.1105/tpc.105.031716 PMC1091760

[CIT0058] Mallory AC Vaucheret H. 2006. Functions of microRNAs and related small RNAs in plants. Nature Genetics 38, S31-–36 16736022 10.1038/ng1791

[CIT0059] Marschner H. 1995. Effect of internal and external factors on root growth and development. In: Marscher H, ed. *Mineral nutrition of higher plants* , 2nd edn. London: Academic Press, 508–536

[CIT0060] Meng Y Chen D Ma X Mao C Cao J Wu P Chen M. 2010. Mechanisms of microRNA-mediated auxin signaling inferred from the rice mutant *osaxr* . Plant Signaling and Behavior 5, 252–254 20023405 10.4161/psb.5.3.10549 PMC2881269

[CIT0061] Meng Y Huang F Shi Q Cao J Chen D Zhang J Ni J Wu P Chen M. 2009. Genome-wide survey of rice microRNAs and microRNA–target pairs in the root of a novel auxin-resistant mutant. Planta 230, 883–898 19655164 10.1007/s00425-009-0994-3

[CIT0062] Mittal D Madhyastha DA Grover A. 2012. Genome-wide transcriptional profiles during temperature and oxidative stress reveal coordinated expression patterns and overlapping regulons in rice. PLoS One 7, e40899 22815860 10.1371/journal.pone.0040899 PMC3397947

[CIT0063] Motose H Hamada T Yoshimoto K Murata T Hasebe M Watanabe Y Hashimoto T Sakai T Takahashi T. 2011. NIMA-related kinases 6, 4, and 5 interact with each other to regulate microtubule organization during epidermal cell expansion in *Arabidopsis thaliana* . The Plant Journal 67, 993–1005 21605211 10.1111/j.1365-313X.2011.04652.x

[CIT0064] Mullen MA Assmann SM Bevilacqua PC. 2012. Toward a digital gene response: RNA G-quadruplexes with fewer quartets fold with higher cooperativity. Journal of the American Chemical Society 134, 812–815 22239732 10.1021/ja2096255

[CIT0065] Nagasaki H Itoh J Hayashi K. 2007. The small interfering RNA production pathway is required for shoot meristem initiation in rice. Proceedings of the National Academy of Sciences, USA 104, 14867–14871 10.1073/pnas.0704339104 PMC1976227 17804793

[CIT0066] Nakajima Y Suzuki S. 2013. Environmental stresses induce misfolded protein aggregation in plant cells in a microtubule-dependent manner. International Journal of Molecular Sciences 14, 7771–7783 23574938 10.3390/ijms14047771 PMC3645715

[CIT0067] Nogueira FT Madi S Chitwood DH Juarez MT Timmermans MC. 2007. Two small regulatory RNAs establish opposing fates of a developmental axis. Genes and Development 21, 750–755 17403777 10.1101/gad.1528607 PMC1838527

[CIT0068] Nozawa M Miura S Nei M. 2012. Origins and evolution of microRNA genes in plant species. Genome Biology and Evolution 4, 230–239 22223755 10.1093/gbe/evs002 PMC3318440

[CIT0069] Overvoorde P Fukaki H Beeckman T. 2010. Auxin control of root development. Cold Spring Harbor Perspectives in Biology 2, a001537 20516130 10.1101/cshperspect.a001537 PMC2869515

[CIT0070] Pant BD Musialak-Lange M Nuc P May P Buhtz A Kehr J Walther D Scheible WR. 2009. Identification of nutrient-responsive Arabidopsis and rapeseed microRNAs by comprehensive real-time polymerase chain reaction profiling and small RNA sequencing. Plant Physiology 150, 1541–1555 19465578 10.1104/pp.109.139139 PMC2705054

[CIT0071] Park MY Wu G Gonzalez-Sulser A Vaucheret H Poethig RS. 2005. Nuclear processing and export of microRNAs in *Arabidopsis* . Proceedings of the National Academy of Sciences, USA 102, 3691–3696 10.1073/pnas.0405570102 PMC553294 15738428

[CIT0072] Pieczynski M Marczewski W Hennig J. 2013. Down-regulation of *CBP80* gene expression as a strategy to engineer a drought-tolerant potato. Plant Biotechnology Journal 11, 459–469 23231480 10.1111/pbi.12032

[CIT0073] Qiao M Zhao Z Song Y Liu Z Cao L Yu Y Li S Xiang F. 2012. Proper regeneration from *in vitro* cultured *Arabidopsis thaliana* requires the microRNA-directed action of an auxin response factor. The Plant Journal 71, 14–22 22335436 10.1111/j.1365-313X.2012.04944.x

[CIT0074] Qin D Wu H Peng H Yao Y Ni Z Li Z Zhou C Sun Q. 2008. Heat stress-responsive transcriptome analysis in heat susceptible and tolerant wheat (Triticum aestivum L.) by using Wheat Genome Array. BMC Genomics 9, 432 18808683 10.1186/1471-2164-9-432 PMC2614437

[CIT0075] Ramakers C Ruijter JM Deprez RH Moorman AF. 2003. Assumption-free analysis of quantitative real-time polymerase chain reaction (PCR) data. Neuroscience Letters 339, 62–66 12618301 10.1016/s0304-3940(02)01423-4

[CIT0076] Raz V Ecker JR. 1999. Regulation of differential growth in the apical hook of *Arabidopsis* . Development 126, 3661–3668 10409511 10.1242/dev.126.16.3661

[CIT0077] Reddy AS Marquez Y Kalyna M Barta A. 2013. Complexity of the alternative splicing landscape in plants. The Plant Cell 25, 3657–3683 24179125 10.1105/tpc.113.117523 PMC3877793

[CIT0078] Reinhart BJ Weinstein EG Rhoades MW Bartel B Bartel DP. 2002. MicroRNAs in plants. Genes and Development 16, 1616–1626 12101121 10.1101/gad.1004402 PMC186362

[CIT0079] Rhoades MW Reinhart BJ Lim LP Burge CB Bartel B Bartel DP. 2002. Prediction of plant microRNA targets. Cell 110, 513–520 12202040 10.1016/s0092-8674(02)00863-2

[CIT0080] Ruiz-Ferrer V Voinnet O. 2009. Roles of plant small RNAs in biotic stress responses. Annual Review of Plant Biology 60, 485–510 10.1146/annurev.arplant.043008.092111 19519217

[CIT0081] Sauer M Robert S Kleine-Vehn J. 2013. Auxin: simply complicated. Journal of Experimental Botany 64, 2565–2577 23669571 10.1093/jxb/ert139

[CIT0082] Savin R Stone PJ Nicolas ME Wardlaw IF. 1997. Grain growth and malting quality of barley. 2. Effects of temperature regime before heat stress. Australian Journal of Agricultural Research 48, 625–634

[CIT0083] Schumann PHD Richardson AE Smith FW Delhaize E. 2004. Characterization of promoter expression patterns derived from Pht1 transporter genes of barley (Hordeum vulgare L.). Journal of Experimental Botany 55, 855–865 15020637 10.1093/jxb/erh103

[CIT0084] Schwab R Palatnik JF Riester M Schommer C Schmid M Weigel D. 2005. Specific effects of microRNAs on the plant transcriptome. Developmental Cell 8, 517–527 15809034 10.1016/j.devcel.2005.01.018

[CIT0085] Schwab R Speth C Laubinger S Voinnet O. 2013. Enhanced microRNA accumulation through stemloop-adjacent introns. EMBO Reports 14, 615–621 23661080 10.1038/embor.2013.58 PMC3701234

[CIT0086] Seki M Umezawa T Urano K Shinozaki K. 2007. Regulatory metabolic networks in drought stress responses. Current Opinion in Plant Biology 10, 296–302 17468040 10.1016/j.pbi.2007.04.014

[CIT0087] Shin C Feng Y Manley JL. 2004. Dephosphorylated SRp38 acts as a splicing repressor in response to heat shock. Nature 427, 553–558 14765198 10.1038/nature02288

[CIT0088] Sunkar R. 2010. MicroRNAs with macro-effects on plant stress responses. Semininars in Cell and Developmental Biology 21, 805–811 10.1016/j.semcdb.2010.04.001 20398781

[CIT0089] Sunkar R Kapoor A Zhu JK. 2006. Posttranscriptional induction of two Cu/Zn superoxide dismutase genes in *Arabidopsis* is mediated by downregulation of miR398 and important for oxidative stress tolerance. The Plant Cell 18, 2051–2065 16861386 10.1105/tpc.106.041673 PMC1533975

[CIT0090] Sunkar R Zhu JK. 2004. Novel and stress-regulated microRNAs and other small RNAs from Arabidopsis. The Plant Cell 16, 2001–2019 15258262 10.1105/tpc.104.022830 PMC519194

[CIT0091] Szarzynska B Sobkowiak L Pant BD Balazadeh S Scheible WR Mueller-Roeber B Jarmolowski A Szweykowska-Kulinska Z. 2009. Gene structures and processing of *Arabidopsis thaliana* HYL1-dependent pri-miRNAs. Nucleic Acids Research 37, 3083–3093 19304749 10.1093/nar/gkp189 PMC2685107

[CIT0092] Tabata R Ikezaki M Fujibe T Aida M Tian CE Ueno Y Yamamoto KT Machida Y Nakamura K Ishiguro S. 2010. Arabidopsis AUXIN RESPONSE FACTOR 6 and 8 regulate jasmonic acid biosynthesis and floral organ development via repression of class 1 *KNOX* genes. Plant and Cell Physiology 51, 164–175 20007966 10.1093/pcp/pcp176

[CIT0093] Vaucheret H Vazquez F Crété P Bartel DP. 2004. The action of *ARGONAUTE1* in the miRNA pathway and its regulation by the miRNA pathway are crucial for plant development. Genes and Development 18, 1187–1197 15131082 10.1101/gad.1201404 PMC415643

[CIT0094] Voinnet O. 2009. Origin, biogenesis, and activity of plant microRNAs. Cell 136, 669–687 19239888 10.1016/j.cell.2009.01.046

[CIT0095] Wahid A Gelani S Ashraf M Foolad MR. 2007. Heat tolerance in plants: an overview. Environmental and Experimental Botany 61, 199–223

[CIT0096] Wallwork MAB Logue SJ MacLeod LC Jenner CF. 1998. Effect of high temperature during grain filling on starch synthesis in the developing barley grain. Australian Journal of Plant Physiology 25, 173–181

[CIT0097] Wang Y Sun F Cao H Peng H Ni Z Sun Q Yao Y. 2012. *TamiR159* directed wheat *TaGAMYB* cleavage and its involvement in anther development and heat response. PLoS One 7, e48445 23133634 10.1371/journal.pone.0048445 PMC3486836

[CIT0098] Xin M Wang Y Yao Y Xie C Peng H Ni Z Sun Q. 2010. Diverse set of microRNAs are responsive to powdery mildew infection and heat stress in wheat (Triticum aestivum L.). BMC Plant Biology 10, 123 20573268 10.1186/1471-2229-10-123 PMC3095282

[CIT0099] Yang L Liu Z Lu F Dong A Huang H. 2006. SERRATE is a novel nuclear regulator in primary microRNA processing in Arabidopsis. The Plant Journal 47, 41–850 10.1111/j.1365-313X.2006.02835.x 16889646

[CIT0100] Yost HJ Lindquist S. 1986. RNA splicing is interrupted by heat shock and is rescued by heat shock protein synthesis. Cell 45, 185–193 2421918 10.1016/0092-8674(86)90382-x

[CIT0101] Yu X Wang H Lu Y de Ruiter M Cariaso M Prins M van Tunen A He Y. 2012. Identification of conserved and novel microRNAs that are responsive to heat stress in *Brassica rapa* . Journal of Experimental Botany 63, 1025–1038 22025521 10.1093/jxb/err337 PMC3254694

[CIT0102] Zadoks JC Chang TT Konzak CF. 1974. A decimal code for the growth stages of cereals. Weed Research 14, 415–421

[CIT0103] Zhang J Xu Y Huan Q Chong K. 2009. Deep sequencing of *Brachypodium* small RNAs at the global genome level identifies microRNAs involved in cold stress response. BMC Genomics 10, 449 19772667 10.1186/1471-2164-10-449 PMC2759970

[CIT0104] Zhao B Ge L Liang R Li W Ruan K Lin H Jin Y. 2009. Members of miR-169 family are induced by high salinity and transiently inhibit the NF-YA transcription factor. BMC Molecular Biology 10, 29 19351418 10.1186/1471-2199-10-29 PMC2670843

