# Supplementary material for: Transcriptionally and post-transcriptionally regulated microRNAs in heat stress response in barley
Source: J Exp Bot. 2014 Sep 2;65(20):6123–35. doi: 10.1093/jxb/eru353 (PMC4203144; doi:10.1093/jxb/eru353)
Supplement: Supplementary Data [file supp_65_20_6123__index.html]

Transcriptionally and post-transcriptionally regulated microRNAs in heat stress response in barley — Transcriptionally and post-transcriptionally regulated microRNAs in heat stress response in barley — Supplementary Data 

# Transcriptionally and post-transcriptionally regulated microRNAs in heat stress response in barley

## Supplementary Data

Data files

**Files in this Data Supplement:**

- Supplementary Data - Supplementary Data
