# Supplementary material for: Transcriptionally and post-transcriptionally regulated microRNAs in heat stress response in barley
Source: J Exp Bot. 2014 Sep 2;65(20):6123–35. doi: 10.1093/jxb/eru353 (PMC4203144; doi:10.1093/jxb/eru353)

Journal of Experimental Botany

Transcriptionally and post-transcriptionally regulated microRNAs in heat stress response in barley

Katarzyna Kruszka, Andrzej Pacak, Aleksandra Swida-Barteczka, Przemyslaw Nuc, Zuzanna Wroblewska, Sylwia Alaba, Wojciech Karlowski, Artur Jarmolowski and Zofia Szweykowska-Kulinska

Table S1 Primers and hybridization probes used in the experiments

primer	sequence (5'→ 3')	miRNA/gene	application
APO131	GTGCTCACTCTCTTCTGCT	156	probe - mature miRNA
KK65	CAGAGCTCCCTTCAATCCAAA	159	probe - mature miRNA
KK397	CTTCTTCCTCCTTCCTCCACCCAC	160a	RT-PCR
KK398	GGATCGACCTGCACCGTGATC	160a	RT-PCR
OS205	CCAAGCATGACCGTCTCTCT	160a	Real time PCR – pri-miRNA
OS206	GATCGGGTTACCCTCTACCA	160a	Real time PCR – pri-miRNA
KK513	TGAAGGTTCGGCAGGAAGAGCTAG	160a	Real time PCR – spliced isoform e2/e3
KK524	ACAAGCACTGTACATATGGGATCGGG	160a	Real time PCR – spliced isoform e2/e3
KK183	AGGCATACAGGGAGCCAGGCA	160	probe - mature miRNA
KK445	AGAGGGTGAGGAACTGAGGATGC	166a	RT-PCR
KK446	GAGAGGAGAGAGAAAGGGAAGACG	166a	RT-PCR
HvReTi_40F	TCTGAGGTCTGAGGATGGAAA	166a	Real time PCR – pri-miRNA
OS187	CCCCCTTACCCAAGAAACAT	166a	Real time PCR – pri-miRNA
KK417	GGAATGAAGCCTGGTCCGAGA	166	probe - mature miRNA
HvReTi_5_F	CAGCGTCATCTTCTTCGTTTC	166n	Real time PCR – pri-miRNA
HvReTi_5_R	AGGTGGAGCTCACAAGAACAC	166n	Real time PCR – pri-miRNA
KK66	GGGGAATGAAGCCTGGTCCGA	166	probe - mature miRNA
KK413	CAATTATTCCCACCTCTCTCTC	167h	RT-PCR
KK414	AATCACAAGCCACAAGATCG	167h	RT-PCR
HvReTi_41F	TTGATGGGTAGATCAAGGTGC	167h	Real time PCR – pri-miRNA
OS203	TGGGATTTTAGGGTTTTCGTT	167h	Real time PCR – pri-miRNA
KK380	TCAGATCATGCTGGCAGCTTCA	167	probe - mature miRNA
KK67	GTCCCGATCTGCACCAAGCGA	168	probe - mature miRNA
KK68	GATATTGGCACGGCTCAATCA	171	probe - mature miRNA
KK69	CCGTTGAGTGCAGCGTTGATG	397	probe - mature miRNA
OS55	AACACACACACACACTGAGCATAG	530-5p	RT-PCR
OS71	TAGCTCCGAAGTTCGCTTGAAGCATGTC	530-5p	RT-PCR
OS114	GTCGAATCAGTGCACAGCAA	530-5p	Real time PCR – pri-miRNA
HvuReTi_45R	ACCTATCGGAAGAGCAAGAGG	530-5p	Real time PCR – pri-miRNA
OS82	GTAGGTGCAGGTGCAAATGCA	530-5p	probe - mature miRNA
KK180	CTCCGTCCCATAATATAAGAATGT	1120	probe - mature miRNA
KK211	GCGAGGAAGAAGGAGAAGAAAG	1120b	RT-PCR
KK212	TCAATCAAACATGGTTAGCATCAGGT	1120b	RT-PCR
OS185	GCTTATCTATGAAACACGTTGAACA	1120b	Real time PCR – pri-miRNA

OS186	AAGAGCGTTTTAAACACAACACTA	1120b	Real time PCR – pri-miRNA
KK220	ACTCCCTCCGTTCCATAATATAAG	1120	probe - mature miRNA
KK182	TTCCGTATGTAGTCCATAGTTGA	1126	probe - mature miRNA
KK482	ACAGAGACAGAGATACCTTTGAG	1432-5p	RT-PCR
KK483	TCATGTAGACGTAAGTCGTCGTC	1432-5p	RT-PCR
HvuReTi_11F	TCTTGGTGGTCTTGTGGGTTA	1432-5p	Real time PCR – pri-miRNA
HvuReTi_11R	AAACGGATACATCATGGCCTA	1432-5p	Real time PCR – pri-miRNA
OS92	GTCGGTGTCATCTCTCCTGAA	1432-5p	probe - mature miRNA
KK466	GCACTACAAACAGGCAGATTCG	5175a	RT-PCR
KK467	CTGGAACCGACACATCTCCTCTC	5175a	RT-PCR; Real time PCR – spliced isoform
HvuReTi_42F	CCTCCCTCCCTTGTAGACTTG	5175a	Real time PCR – pri-miRNA
HvuReTi_42R	CCGATAGCTGTCATTAACCCA	5175a	Real time PCR – pri-miRNA
KK484	ACCACACGCCAAAACAGGGAG	5175a	Real time PCR – spliced isoform
KK454	TCCCTCCGTCCCAAAATTCTT	5175	probe - mature miRNA
KK468	CACAACAGGAACACCTTGGTC	5203	RT-PCR
KK469	CAGAAAAAACACCATGTTCGTGCTG	5203	RT-PCR
OS188	CGGACCATGGACAAACTACT	5203	Real time PCR – pri-miRNA
OS189	AAAACACTCGCTCGCTCCTA	5203	Real time PCR – pri-miRNA
KK456	TCCCTCCGTTCCAAAATAAGT	5203	probe - mature miRNA
KK531	CATCTGGGAGGGGTTGACATGAGTTCCAGC	PHV	5’ RACE
KK532	GGAGTGTTAGGTCCTGATGGACCCCCAGTGG	PHV	5’ RACE
KK511	GTGGCGACTACAGACACAAGC	PHV	Real time PCR
KK512	CGGAACTACTGCGTTCTGCTGC	PHV	Real time PCR
KK493	CGCTGGAGCGGGTGTATGCC	REV	Real time PCR
KK494	TCTGCCTGGGCTCGATGTTGGAC	REV	Real time PCR
KK546	CCGATGCCTGGGATGAAGCCT	HOX9	Real time PCR
KK547	CTAGATTCACCAGACCACAGGCACG	HOX9	Real time PCR
KK537	GGTTTAGCCAGCCCACCCTTGTCGCCAC	ARF17	5’ RACE
KK538	ACCTTGCAGTGCCCAGCCTCCAGTCCGAGGTC	ARF17	5’ RACE
KK487	AGCTCTCCTCCTTCGCCAAGACG	ARF17	Real time PCR
KK520	GTAGATGTGCCGGAACTTCCACACC	ARF17	Real time PCR
KK548	TCCTCTCGCAGGGATACAGGG	ARF13	Real time PCR
KK549	GGAGTAGAGCAGGCTCGACTGC	ARF13	Real time PCR
KK489	TGGAGGATGGAGGACCTGCTCTG	ARF8	Real time PCR
KK490	TGCGGCATCTCGGACATGGAG	ARF8	Real time PCR
KK541	ACGTTGGGCTTCAGAGCGGCAGAACC	Nek5-like kinase	5’ RACE
KK542	TTCCCTGTTACTGCCTGCTCACCACTGACAC	Nek5-like kinase	5’ RACE
KK497	CTAGGACGTGGAGCTGATCGG	Nek5-like kinase	Real time PCR
KK498	CTCGTATTGATCCATCCGTGACTCC	Nek5-like kinase	Real time PCR
APO395	AATAACGTGTTTTGGGCAAACTT	HvPht1-1	cDNA purity control
APO396	AAGGGACATTTCCTCGCTACTTG	HvPht1-1	cDNA purity control

APO387	CGTGACGCTGTGTTGCTTGT	ADP	Real time PCR
APO388	CCGCATTCATCGCATTAGG	ADP	Real-time-PCR
UBQF	CCTGCGTGGTGGCAAGTAAG	UBQ	RT-PCR
UBQR	ACAACCAGACATGCTCCAACCT	UBQ [86]	RT-PCR
U6	TCATCCTTGCGCAGGGGCCA	U6	probe
KK435	CCCTTCTCGCTCGACCTCTG	HSP17	RT-PCR
KK436	GGTGATCTGGACGGGCTTGAC	HSP17	RT-PCR

Supplementary Fig. S1

Schematic representation of barley *MIR* genes and the hairpin structures of barley/plant ortholog of the pre-miRNA precursors.

A-H The structure of barley *MIR* genes; miRNA and miRNA* are marked in red and blue, respectively; the black vertical lines represent the start and stop codons of the ORF for protein-coding genes containing miRNAs; lines above the gene structures represent the alternative splicing events.
Below the structures of the *MIR* genes pre-miRNA hairpin structures for barley and its plant orthologue are presented. For each hairpin structure the lowest minimal folding free energy (ΔG kcal/mol) is included.
hvu – *Hordeum vulgare*; osa – *Oryza sativa*; tae – *Triticum aestivum*; bdi – *Brachypodium distachyon*.

A *MIR166a gene*

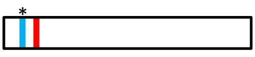

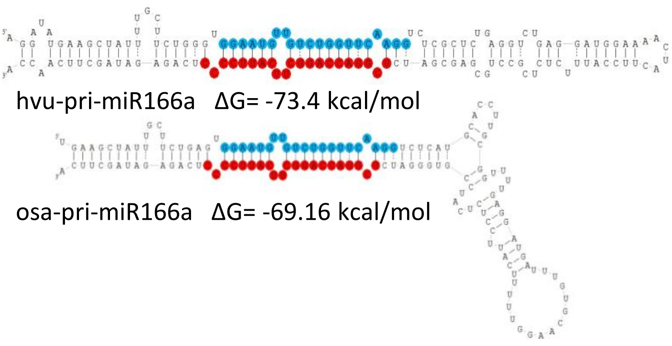

B *MIR167h* gene

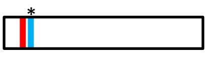

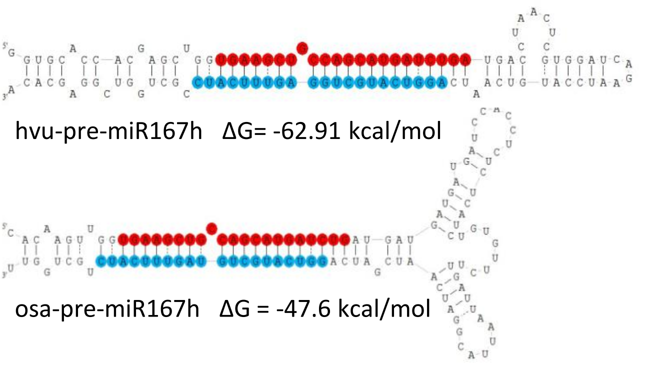

C *MIR530-5p* gene

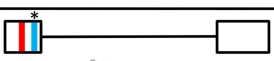

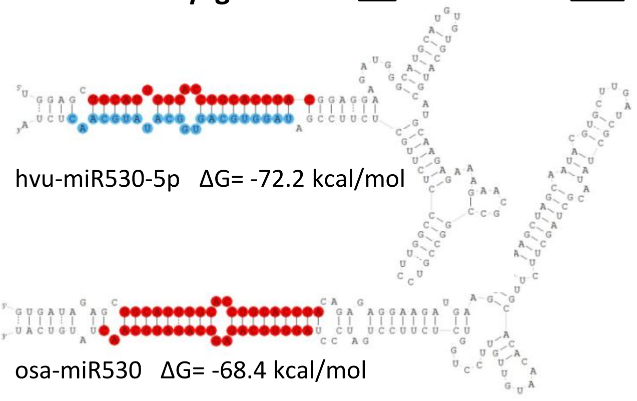

D *MIR1432-5p* gene

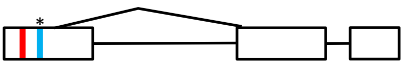

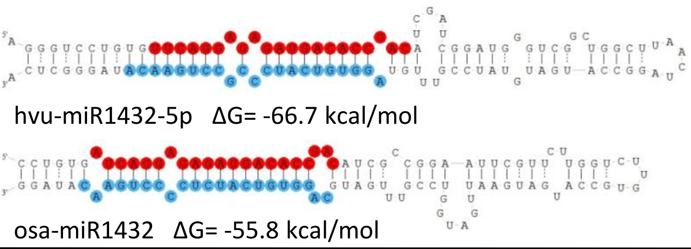

E *MIR160a* gene

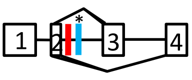

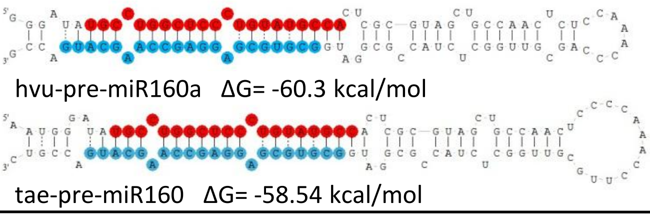

F *30S ribosomal protein S16-like/MIR1120b* gene

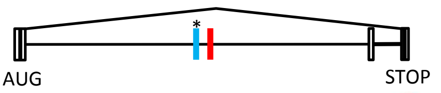

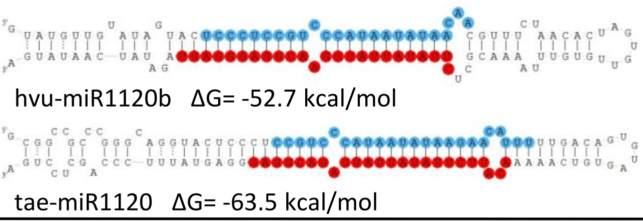

G *RNA pol II phoshatase-like/MIR5175a* gene

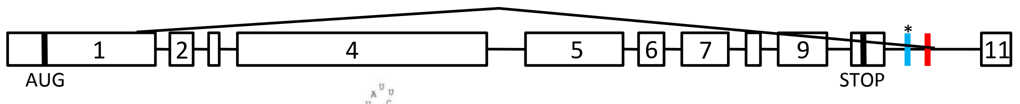

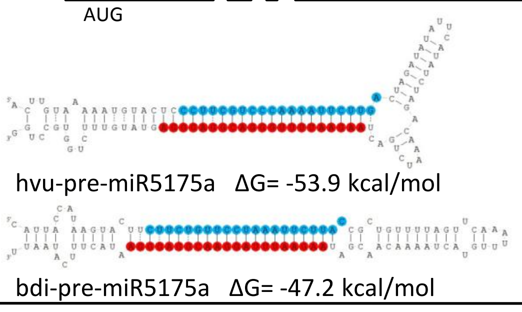

H *ATP-dependent Clp protease proteolytic subunit-related protein/MIR5203* gene

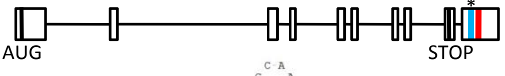

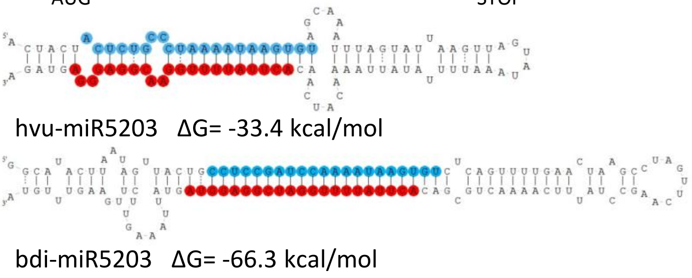

Supplementary Fig. S2

The expression profiles at the level of pri-miRNAs and mature miRNAs during barley development.

A-H Left panels present RT-PCR analysis of the expression level of barley pri-miRNAs; primer positions are marked by black triangles on the pri-miRNA schematic structures; ubiquitin (UBQ) amplification was used as a loading control.
Middle panels show qRT-PCR measurements of the expression level of pri-miRNAs; bars represent the means of three independent biological samples ± SE.
Right panels display the mature miRNAs detection by Northern hybridization; U6 was used as a loading control; the level of miRNA in 1-week-old plants (with the exception of miR5175, where the miR level in 2-week-old plants was chosen) was arbitrarily assumed to be '1', and the levels of miRNA were quantified relative to this at all other growth stages tested.
Abbreviations: 1w: one-week-old seedlings, 2w: two-week-old seedlings, 3w: three-week-old plants, 6w: six-week-old plants, 68d: 68-day-old plants, gD: genomic DNA, -: negative control lacking template, M - Gene Ruler 100 bp Plus or 1kb Plus DNA Ladders, kb: kilobase pair.

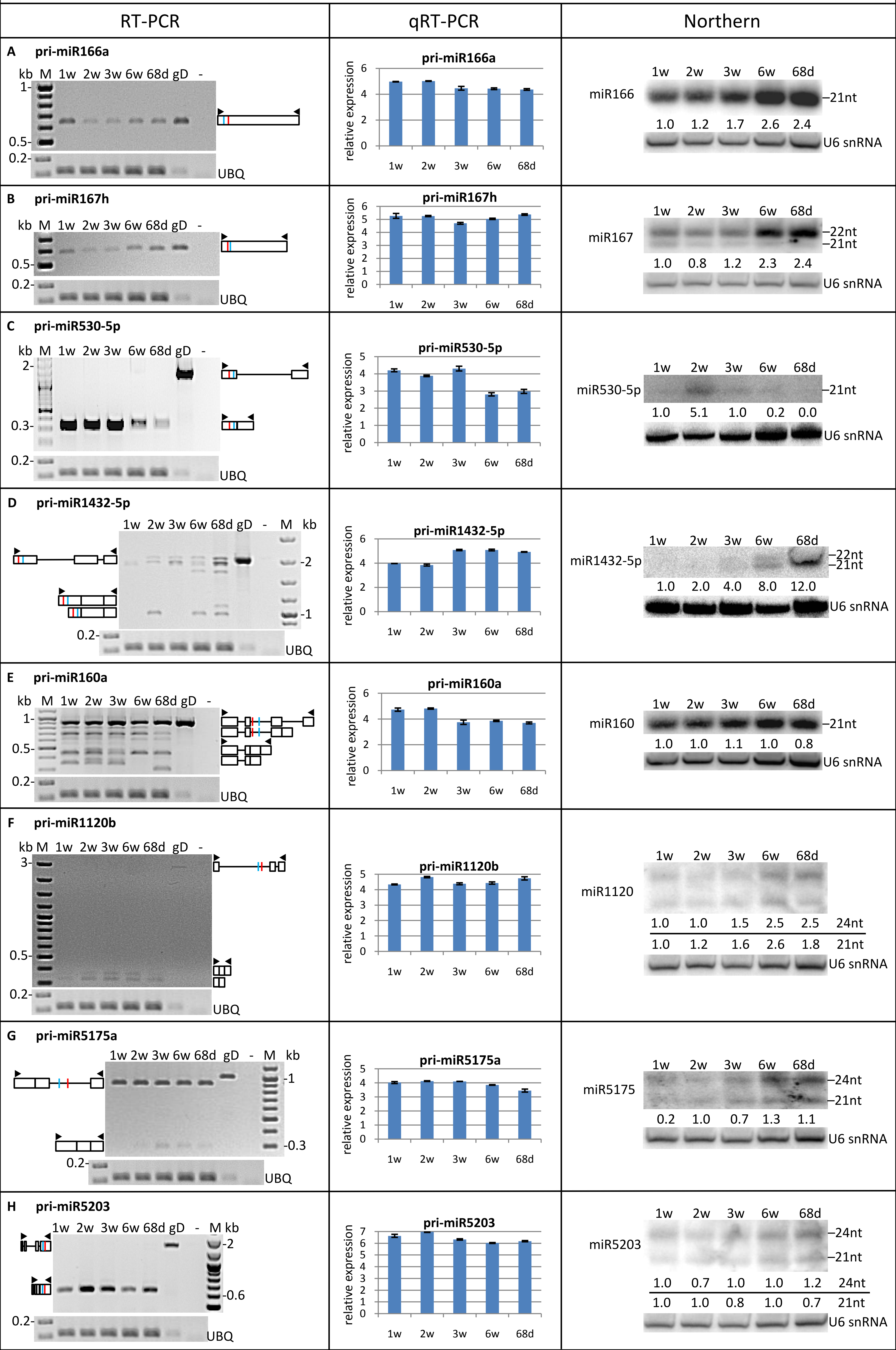

Supplementary Fig. S3

The level of mature barley miRNAs under heat stress

Mature miRNAs miR156, 159, 166, 168, 171 and 397 (A-F, respectively) were detected by Northern hybridization in control and heat conditions. The level of mature miRNAs was analyzed at different time points: 3, 6 and 24 hours. U6 was used as a loading control. The level of miRNA in heat stress was quantified relative to the one in control condition in respective time-points tested.

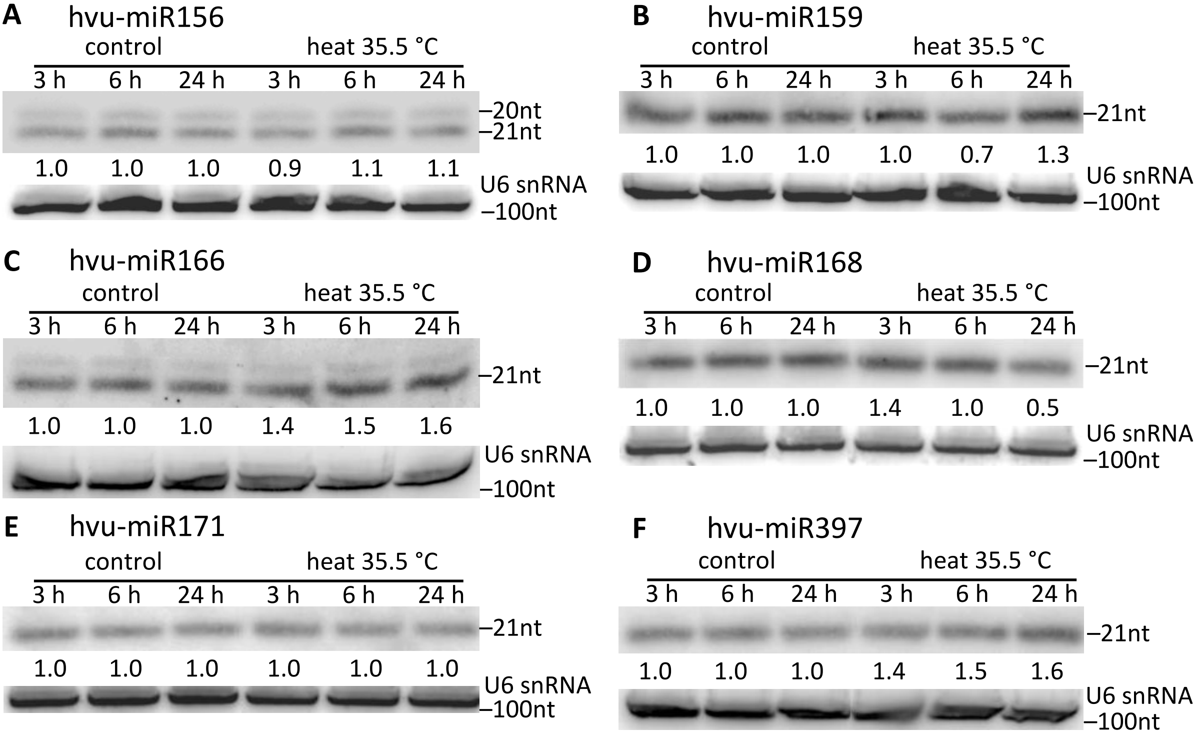

Supplementary Fig. S4

Expression of the heat stress marker gene *HSP17* in barley plants subjected to heat stress.

We analysed the expression level of barley ortholog of *ATHSP17.4* heat shock protein gene from *Arabidopsis thaliana* to confirm the response of the barley plants during heat stress. The expression analysis was performed at different time points: 3, 6 and 24 hours in control and heat stress conditions. Ubiquitin (UBQ) amplification was used as loading control. Abbreviations: -: negative control lacking template, M - Gene Ruler 100 bp Plus, kb: kilobase pair.

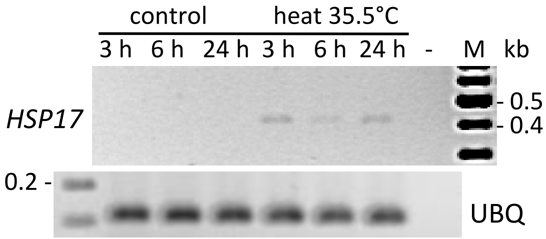

Supplement: Supplementary Data [file supp_eru353_jexbot126110_file001.pdf]
